# Estimation of Short-Term Effects of Air Pollution on Stroke Hospital Admissions in Wuhan, China

**DOI:** 10.1371/journal.pone.0061168

**Published:** 2013-04-12

**Authors:** Hao Xiang, Kristen J. Mertz, Vincent C. Arena, Luann L. Brink, Xiaohui Xu, Yongyi Bi, Evelyn O. Talbott

**Affiliations:** 1 Department of Epidemiology and Biostatistics, School of Public Health, Wuhan University, Wuhan, Hubei, China; 2 Wuhan University Global Health Institute, Wuhan University, Wuhan, Hubei, China; 3 Department of Epidemiology, Graduate School of Public Health, University of Pittsburgh, Pittsburgh, Pennsylvania, United States of America; 4 Department of Biostatistics, Graduate School of Public Health, University of Pittsburgh, Pittsburgh, Pennsylvania, United States of America; 5 Department of Epidemiology, College of Public Health and Health Professions, College of Medicine, University of Florida, Gainesville, Florida, United States of America; 6 Department of Labor and Environmental Hygiene, School of Public Health, Wuhan University, Wuhan, Hubei, China; Virginia Commonwealth University, United States of America

## Abstract

**Background and Objective:**

High concentrations of air pollutants have been linked to increased incidence of stroke in North America and Europe but not yet assessed in mainland China. The aim of this study is to evaluate the association between stroke hospitalization and short-term elevation of air pollutants in Wuhan, China.

**Methods:**

Daily mean NO_2_, SO_2_ and PM_10_ levels, temperature and humidity were obtained from 2006 through 2008. Data on stroke hospitalizations (ICD 10: I60–I69) at four hospitals in Wuhan were obtained for the same period. A time-stratified case-crossover design was performed by season (April-September and October-March) to assess effects of pollutants on stroke hospital admissions.

**Results:**

Pollution levels were higher in October-March with averages of 136.1 µg/m^3^ for PM_10_, 63.6 µg/m^3^ for NO_2_ and 71.0 µg/m^3^ for SO_2_ than in April-September when averages were 102.0 µg/m^3^, 41.7 µg/m^3^ and 41.7 µg/m^3^, respectively (*p*<.001). During the cold season, every 10 µg/m^3^ increase in NO_2_ was associated with a 2.9% (95%C.I. 1.2%–4.6%) increase in stroke admissions on the same day. Every 10 ug/m^3^ increase in PM_10_ daily concentration was significantly associated with an approximate 1% (95% C.I. 0.1%–1.4%) increase in stroke hospitalization. A two-pollutant model indicated that NO_2_ was associated with stroke admissions when controlling for PM_10_. During the warm season, no significant associations were noted for any of the pollutants.

**Conclusions:**

Exposure to NO_2_ is significantly associated with stroke hospitalizations during the cold season in Wuhan, China when pollution levels are 50% greater than in the warm season. Larger and multi-center studies in Chinese cities are warranted to validate our findings.

## Introduction

Numerous epidemiological studies have demonstrated increased risk for acute myocardial infarction (MI), coronary heart disease (CHD) and other cardiovascular mortality associated with acute exposure to ambient air pollutants [1]–[4]. More recently, studies have focused on morbidity with use of hospital admission data to assess the short-term effects of air pollution on stroke; however, the results of these studies are inconsistent. Some studies have found associations between air pollution and stroke hospital admissions [5]–[11], while other studies have reported no significant links [12]–[16]. Moreover, most studies have been conducted in North American and European cities [5]–[7], [9], [13], [14], [17] with only limited information from Asia [8], [12], [18].

Wuhan is the capital of Hubei Province and the most populous city in Central China. There are approximately 10 million people in the metropolitan area, with approximately 6 million in the center city within an area of 201 km^2^. Wuhan’s climate is subtropical with abundant rainfall and very high humidity in the spring and summer. Wuhan’s major industries include optic-electronics, automobile manufacturing, steel manufacturing, chemical plants and power plants; it is also a major transportation hub.The major sources of air pollution in the city are motor vehicles and the use of coal for domestic cooking, heating, and industrial processes [19], [20].

Qian et al’s study in Wuhan showed that every 10 µg/m^3^ increase in daily PM_10_ concentration was significantly associated with an increase in stroke mortality (0.44%) on the same day [20]; however, the effect of air pollution on stroke hospital admissions has not been assessed in Wuhan, which has a hotter climate and higher levels of pollutants than most places studied to date. The objective of this study is to assess the associations between hospitalizations for stroke and short-term elevations in particulate matter with diameter <10 µm (PM_10_), nitrogen dioxide (NO_2_) and sulfur dioxide (SO_2_), in Wuhan, China. The research covers a 3-year period from 2006 through 2008.

## Materials and Methods

### Ethics Statement

This study was approved by the Institutional Review Boards of Wuhan University and the University of Pittsburgh. Because the data were analyzed anonymously, names of participants were not essential, and we did not collect the names of participant. Informed consent from human participant was not necessary in this study.

### Study Population

Hospital admission data for this analysis were collected from four major medical centers with a total of 10,000 beds in the center city because there is no unified hospital data collection system for all hospitals in Wuhan, We obtained electronic data for all hospital admissions for acute strokes (International Classification of Diseases, 10th Revision: I60–I69 ) from January 1, 2006 through December 31, 2008, including admission date, age, sex, and discharge diagnostic code (ICD-10).

### Exposure Information

Nine monitoring stations operated by the Wuhan Environmental Monitoring Center in the metropolitan area measure levels of air pollution. The monitoring program has been certified by the US Environment Protection Agency (EPA). We obtained the mean daily Air Pollution Index (API) of PM_10_, NO_2_ and SO_2_ for the 9 monitoring stations for the period January 1, 2006 through December 31, 2008. API reporting system, based on health effects, is introduced for a consistent comparison among the pollution levels by different air pollutants. In China, the API system has been applied in cities from 1997 [21]. Other similar index systems are also used in other countries, for example, AQI (Air Quality Index) in Canada, IMECA (Air Quality Metropolitan Index) in Mexico and PSI (Pollutant Standards Index) in Singapore. According to the breakpoints of air pollutant and the transform formula between API and air pollutant concentrations. We converted the API values to concentration levels using standard formula [22]. Meteorological data, including mean daily temperature and relative humidity, were obtained from the Wuhan Meteorological Administration.

### Data Analysis

A time-stratified case-crossover study design was used to evaluate the association between air pollution and the number of stroke admissions. This method is a special case-control design used to explore the effects of transient exposures on acute events, in which every case serves as his or her own control [22], [23]. We compared the air pollution exposure on the current day (lag 0) of the stroke onset to exposure on the same weekdays during the same month as the lag 0 day. Because previous studies indicated that increased hospital admissions were associated with high air pollution levels up to 2 days before the event [24], we also considered pollution concentrations one or two days before stroke admission (lag1, lag2) and the accumulated exposure over 3 days (mean of lag 0 through lag 2). This design is useful for controlling for seasonality, time trends, and other potential confounders [25].

We performed multivariate conditional logistic regression modeling to obtain estimates of the odds ratios (ORs) and 95% confidence intervals (CIs) for the effect of PM_10_, NO_2_ and SO_2_ on stroke hospitalization, controlling for meteorological covariates including daily mean temperature and relative humidity. For understanding the mean effect of air pollution during lag 0–2 day, we calculated lag 0–2 with available data, we reported effect estimates as the percentage of change in the rate of hospitalizations associated with a 10 µg/m^3^ increase in mean daily pollutant levels. Subgroup analysis is based on age group (≥65 years and <65 years), sex (male and female), categories of stroke (hemorrhagic stroke: I61 and ischemic stroke: I63) and seasons (warm: April-September and cold: October-March). All of the reported *p*-values are based on 2-sided tests at the 0.05 level. Analyses were performed using SAS Version 9 and the C-CAT package [26].

## Results

We observed 10663 hospital admissions with a primary discharge diagnosis of stroke from four major hospitals, of which 51% (n = 5422) were coded as ischemic, 21% (n = 2225) as hemorrhagic, and 28% (n = 3016) as other. Age on admission ranged from 0 to 98 years with a mean of 64(S.D.14) years, and 51% (n = 5480) of those admitted were equal or older than 65 years. Approximately 61% of cases were male.

A summary of air pollutants and meteorological parameters is shown in Table 1. During the 1096 days of our study period, daily mean temperatures fluctuated between −2.7 and 33.6°C. The overall mean concentrations of pollutants were 119±53 µg/m^3^ for PM_10_, 53±21 µg/m^3^ for NO_2_, and 56±31 µg/m^3^ for SO_2_. There were significant differences(*P*<0.001) in pollution levels by season with the April through September means of 102.0 µg/m^3^ for PM_10_, 41.7 µg/m^3^ for NO_2_ and 41.7 µg/m^3^ for SO_2;_ and the October through March means of 136.1 µg/m^3^, 63.6 µg/m^3^ and 71.0 µg/m^3^, respectively. (Table 1).

**Table 1:** Ambient air pollution concentrations and meteorological parameters in Wuhan, 2006–2008.

	Mean	Min.	Q1*	Median	Q3*	Max.	Apr.-Sep. Median (n = 549 days)	Oct.-Mar. Median (n = 547 days)
Pollutants								
PM_10_(µg/m^3^)	119	35	78	110	148	350	94†	132
NO_2_(µg/m^3^)	53	18	35	48	66	154	38†	66
SO_2_(µg/m^3^)	56	8	33	50	72	267	38†	61
Meteorology								
Temperature (°C)	18.2	−2.7	9.9	19.6	26.3	33.6	26.2	10.0
Humidity (%)	69.6	21.0	61.0	70.5	78.0	97.0	68.0	73.0

* Q1, first quartile value; Q3, third quartile value.

† There are significant differences in PM_10_, NO_2_, SO_2_ concentration in summer/fall and winter/spring, Wilcoxon test *P*<0.001.

As shown in Table 2, none of the single-pollutant models with lag 0, lag 1 and lag 2 were associated with hospital admissions for stroke, ischemic stroke or hemorrhagic stroke with all seasons combined. Subgroup analysis by age and sex found no significant associations.

**Table 2:** Association of all strokes, ischemic and hemorrhagic strokes and pollutant concentrations by lag days for 10 µg/m^3^ increases in pollutant levels adjusted for temperature and humidity, Wuhan, 2006–2008.

	Stroke N = 10663		Ischemic Stroke N = 5422		Hemorrhagic Stroke N = 2225	
Lag days	O.R. (95%C.I.)	*P* Value	O.R. (95%C.I.)	*P* Value	O.R. (95%C.I.)	*P* Value
PM_10_						
0	0.997(0.989,1.005)	0.4350	0.999(0.992,1.006)	0.7394	0.993(0.982,1.004)	0.2265
1	0.997(0.990,1.005)	0.5189	1.000(0.993,1.007)	0.8932	0.998(0.987,1.008)	0.6485
2	0.996(0.988,1.004)	0.3134	1.007(0.992,1.005)	0.6358	0.999(0.989,1.009)	0.8663
0–2	0.995(0.985,1.005)	0.3048	1.005(0.990,1.007)	0.7016	0.995(0.983,1.008)	0.4706
NO_2_						
0	0.997(0.977,1.018)	0.7902	1.002(0.984,1.021)	0.8267	0.989(0.961,1.018)	0.4421
1	0.994(0.973,1.015)	0.5703	1.003(0.969,1.006)	0.1849	1.007(0.978,1.036)	0.6403
2	1.010(0.989,1.031)	0.3566	1.000(0.982,1.019)	0.9704	1.004(0.976,1.032)	0.7990
0–2	1.004(0.977,1.031)	0.7703	1.004(0.973,1.018)	0.6672	1.000(0.965,1.035)	0.9841
SO_2_						
0	1.001(0.986,1.016)	0.8792	0.996(0.983,1.009)	0.5549	0.996(0.976,1.016)	0.6773
1	0.992(0.978,1.006)	0.2824	0.994(0.982,1.007)	0.3740	1.003(0.984,1.022)	0.7943
2	0.991(0.977,1.005)	0.1989	0.996(0.984,1.009)	0.5825	0.991(0.973,1.011)	0.3784
0–2	0.991(0.972,1.010)	0.3587	0.993(0.977,1.009)	0.3922	0.995(0.971,1.019)	0.6658

The associations between air pollutants and stroke hospitalization by season are presented in Table 3. For the cold season, significant associations were found between stroke hospital admissions and levels of PM_10,_ and NO_2_. Every 10 µg/m^3^ increase in NO_2_ was associated with a 2.9% (95%C.I. 1.2%–4.6%) increase in stroke events on the same day and a 2.1% increase 2 days later. In addition, each 10 µg/m^3^ increase in PM_10_ daily concentration was significantly associated with an approximate 0.7% (95% C.I. 0.1%–1.4%) increase in stroke hospitalization the next day. SO_2_ levels had no significant effect on stroke hospitalization (Table 3). For the warm season, a time when the pollution levels were lower, no significant associations with stroke hospitalization were noted for any of the pollutants controlling for temperature and humidity.

**Table 3:** The association of stroke admissions and air pollutants in cold (winter and spring) and warm (summer and fall) seasons for 10 µg/m^3^ increases in pollutant level, adjusted for temperature and humidity, Wuhan, 2006–2008.

LagDays	Cold seasons N = 5341		Warm seasons N = 5322	
	O.R. (95%C.I.)	*P* Value	O.R. (95%C.I.)	*P* Value
PM_10_				
0	1.006(0.999,1.013)	0.0875	1.000(0.999,1.000)	0.3174
1	1.007(1.001,1.014)	0.0300	0.995(0.988,1.002)	0.1442
2	1.005(0.999,1.012)	0.0830	0.996(0.989,1.002)	0.1860
0–2	1.009(1.001,1.016)	0.0256	0.994(0.985,1.002)	0.1555
NO_2_				
0	1.029(1.012,1.046)	0.0007	0.998(0.980,1.016)	0.8207
1	1.012(0.995,1.029)	0.1620	0.983(0.965,1.001)	0.0681
2	1.021(1.005,1.037)	0.0096	0.996(0.978,1.014)	0.6584
0–2	1.031(1.011,1.052)	0.0025	0.990(0.967,1.014)	0.4096
SO_2_				
0	1.004(0.993,1.015)	0.4677	0.997(0.984,1.010)	0.6519
1	0.996(0.986,1.007)	0.4929	0.988(0.976,1.001)	0.0621
2	0.997(0.986,1.007)	0.5258	0.994(0.981,1.006)	0.3251
0–2	0.997(0.983,1.011)	0.6844	0.987(0.970,1.004)	0.1307

A two-pollutant model was examined to determine which individual pollutant might influence stroke occurrence independently of the effect of the other (Table 4).

**Table 4:** Relationship between stroke and PM_10_ and NO_2_ concentrations in a two-pollutant model in the cold season for 10 µg/m^3^ increases in pollutant level, adjusted for temperature and humidity.

Pollutant	O.R. (95%CI)	P
PM_10_		
0	0.995(0.986,1.005)	0.3305
1	1.008(0.999,1.017)	0.0931
2	0.998(0.989,1.008)	0.7333
0–2	0.999(0.987,1.011)	0.8945
NO_2_		
0	1.037(1.013,1.061)	0.0021
1	0.997(0.973,1.021)	0.7955
2	1.024(1.000,1.049)	0.0496
0–2	1.033(1.001,1.065)	0.0412

SO_2_ was not included in this model because it was not significantly associated with hospital admissions for stroke. For the cold season, there was no significant association between PM_10_ and stroke admissions after adjusting for NO_2_; however, the effect of NO_2_ remained significant after adjusting for PM_10_.

## Discussion

This is one of the few investigations of the association between short-term health effects and air pollution in mainland China. Single-pollutant models, when adjusted for temperature and humidity, indicated a significant effect from NO_2_ and PM_10_ on stroke hospital admissions during the cold season. The two-pollutant model showed that the effect of NO_2_ remained significant after adjusting for PM_10_.

It should be noted that hospital admissions for hemorrhagic and ischemic strokes, when analyzed separately, were not associated with levels of any air pollutants in single-pollutant models in our study. Given that there were only 2, 225 hemorrhagic and 5, 422 ischemic stroke admissions during the three–year period, we believe that there may be a lack of statistical power to identify such a risk.

Seasonal effects between air pollution and hospital admissions have been reported frequently, and various explanations have been postulated. In the present study, we found that during the warm season, no significant associations were obtained. In contrast, for the cold season of October through March, stroke admissions and PM_10_ and NO_2_ levels were significantly associated. One explanation is that the concentrations of pollutants during the warm season were much lower than in the cold season; the association between air pollution and stroke hospitalization is stronger when pollution levels are higher.

There are three main reasons for higher air pollution levels in the cold season in Wuhan. First, the amount of rainfall decreases significantly in the cold season. The average precipitation in November, December, and January were 2.04, 1.02, 1.71 inches respectively, in contrast to the average rainfall in May, June, and July of 6.47, 8.86, 7.49 inches. Rainfall plays an important role in chemical reactions with air pollutants, diluting their effects. [27] Second, Wuhan was almost controlled by stagnant air and there is less wind in winter than in summer, which went against pollutants diffusion. The formation of inversion layer also made air suspended particles hard to diffuse to high altitude but close to the ground. Third, coal emissions are higher on cold days because of the greater use of coal for electricity and heating. Under extreme conditions, in relative high temprature and low humidity days in winter, air pollutants could be transferred into secondary aerosol and lead to more serious pollution, even formed haze or smog.

It is widely accepted that there is a significant association between mortality and PM_10_ levels. [5], [6], [8] Recent studies showed that air particles maybe a possible causal factor in stroke mortality [28] [29], [30]. Several potential mechanisms have been proposed. First, it has been hypothesized that particulate exposure can induce an acute systemic inflammatory response with an increased number of circulating neutrophils [28], provoking inflammation and increased blood coagulation. [29] Second, particles may affect the autonomic nervous system resulting in increased risk for heart rate variability. [30] Third, particles can increase concentrations of fibrinogen [31] and enhance peripheral arterial thrombosis [32], which will promote stroke. Exposure to cold days has also been proved to increase plasma viscosity and serum cholesterol levels, which is also regarded as a stimulus to stroke [33].

In this study, PM_10_ was significantly associated with stroke hospitalizations in the cold season in a single pollutant model, but not after adjusting for NO_2_. The reason why our result is inconsistent with the past studies may be due to the problem of co-linearity between air pollutants (the pearson correlation between PM_10_ and NO_2_ is 0.75, see supplement Table S1), it was difficult to separate the effect of individual pollutants on stroke admission. Haidong Kan et al’s study showed that in single air pollutant models, each 10 µg/m^3^ increase in PM_10_ and NO_2_ corresponded to 1.008 and 1.029 relative risks of stroke mortality, respectively, but in multiple pollutant models, there were no significant associations [34].

The importance of NO_2_ as a cause of increased stroke admissions is not sufficiently understood. We cannot rule out the possibility that NO_2_ acts, at least in part, as a surrogate for some other unmeasured end product of reaction sequences initiated by NO_2_.

The strengths of our study include the fact that data were derived from the electronic data of four major medical centers, which provided relative accurate information and allowed us to stratify the effect of air pollution by age, sex and stroke type. A further strength of our study was the availability of data on PM_10_, SO_2_ and NO_2_ levels from Wuhan Environmental Monitoring Center, which could be linked for the first time to stroke admissions in Wuhan city. Finally, the case-crossover studies design control for confounders by design rather than by modeling [21], [35]. As a result, time-invariant variables such as sex, age and family history do not act as confounders. In addition, by using the same weekdays as the onset day as controls could avoid possible confounding from the effects of day of the week [22], [36].

We do acknowledge that our study has several limitations. First, there is no unified hospital data collection system for all hospitals in Wuhan, and it is not possible to obtain data on all the stroke admissions in the city during the study period. However, the catchment area of these four hospitals is relatively stable. Second, we used the mean air pollutant concentrations from nine monitoring stations to estimate individuals’ exposure because the air pollutants are highly correlated in 9 monitors. However, errors in exposure estimation resulting from the differences between the population-average exposure and local ambient levels cannot be avoided. Such exposure misclassification may lead to inaccurate results, as reported in previous studies [37], [38]. Third, it should also be noted that Wuhan has a subtropical humid monsoon climate, and this fact may restrict the generalization of these findings to other locations with different meteorological characteristics. However, this would not affect the study’s internal validity.

In summary, our study shows that there is an association between exposure to air pollution and hospital admissions for stroke during the cold season in Wuhan, China. Although these findings support the possibility that there are acute pathogenetic processes in the cerebrovascular system induced by air pollution, the mechanism is still not clear and toxicological analyses are expected in further studies.

## Supporting Information

Table S1 **Pearson correlation coefficient.** This table described correlation coefficient among daily air pollution and weather variables.
(DOC)
