# Supplementary material for: Estimation of Short-Term Effects of Air Pollution on Stroke Hospital Admissions in Wuhan, China
Source: PLoS One. 2013 Apr 12;8(4):e61168. doi: 10.1371/journal.pone.0061168 (PMC3625157; doi:10.1371/journal.pone.0061168)
Supplement: Table S1 — Pearson correlation coefficient. This table described correlation coefficient among daily air pollution and weather variables. (DOC) [file pone.0061168.s001.doc]

**Table S1. Pearson correlation coefficients among daily air pollution and weather variables**

|  | PM_10_ | SO_2_ | NO_2_ | Temperature | Relative Humidity |
| --- | --- | --- | --- | --- | --- |
| PM_10_ | 1.00 |  |  |  |  |
| SO_2_ | 0.68 | 1.00 |  |  |  |
| NO_2_ | 0.75 | 0.69 | 1.00 |  |  |
| Temperature | -0.32 | -0.30 | -0.39 | 1.00 |  |
| Relative Humidity | -0.34 | -0.41 | -0.24 | 0.22 | 1.00 |

**Table 2. Pearson correlation analysis of PM_10_ concentration in the 9 monitors in Wuhan city from 2006 to 2008**

|  | 1^#^ | 2 | 3 | 4 | 5 | 6 | 7 | 8 | 9 |
| --- | --- | --- | --- | --- | --- | --- | --- | --- | --- |
| 1 | 1.00 |  |  |  |  |  |  |  |  |
| 2 | 0.85 | 1.00 |  |  |  |  |  |  |  |
| 3 | 0.88 | 0.83 | 1.00 |  |  |  |  |  |  |
| 4 | 0.85 | 0.87 | 0.80 | 1.00 |  |  |  |  |  |
| 5 | 0.92 | 0.82 | 0.84 | 0.83 | 1.00 |  |  |  |  |
| 6 | 0.92 | 0.85 | 0.86 | 0.86 | 0.91 | 1.00 |  |  |  |
| 7 | 0.90 | 0.90 | 0.84 | 0.89 | 0.88 | 0.90 | 1.00 |  |  |
| 8 | 0.95 | 0.86 | 0.89 | 0.85 | 0.90 | 0.90 | 0.90 | 1.00 |  |
| 9 | 0.92 | 0.89 | 0.87 | 0.89 | 0.9 | 0.93 | 0.92 | 0.91 | 1.00 |

#: 1~9 represent nine monitor sites in urban area of Wuhan city.

**Table 3. Pearson correlation analysis of SO_2_ concentration in the 9 monitors in Wuhan city from 2006 to 2008**

|  | 1 | 2 | 3 | 4 | 5 | 6 | 7 | 8 | 9 |
| --- | --- | --- | --- | --- | --- | --- | --- | --- | --- |
| 1 | 1.00 |  |  |  |  |  |  |  |  |
| 2 | 0.65 | 1.00 |  |  |  |  |  |  |  |
| 3 | 0.66 | 0.85 | 1.00 |  |  |  |  |  |  |
| 4 | 0.72 | 0.75 | 0.65 | 1.00 |  |  |  |  |  |
| 5 | 0.74 | 0.75 | 0.69 | 0.66 | 1.00 |  |  |  |  |
| 6 | 0.83 | 0.74 | 0.72 | 0.72 | 0.73 | 1.00 |  |  |  |
| 7 | 0.73 | 0.81 | 0.70 | 0.72 | 0.66 | 0.69 | 1.00 |  |  |
| 8 | 0.73 | 0.78 | 0.69 | 0.81 | 0.68 | 0.74 | 0.78 | 1.00 |  |
| 9 | 0.82 | 0.68 | 0.71 | 0.75 | 0.72 | 0.78 | 0.77 | 0.77 | 1.00 |

**Table 4. Pearson Correlation Analysis of NO_2_ concentration f in the 9 monitors in Wuhan city rom 2006 to 2008**

|  | 1 | 2 | 3 | 4 | 5 | 6 | 7 | 8 | 9 |
| --- | --- | --- | --- | --- | --- | --- | --- | --- | --- |
| 1 | 1.00 |  |  |  |  |  |  |  |  |
| 2 | 0.65 | 1.00 |  |  |  |  |  |  |  |
| 3 | 0.64 | 0.65 | 1.00 |  |  |  |  |  |  |
| 4 | 0.74 | 0.66 | 0.70 | 1.00 |  |  |  |  |  |
| 5 | 0.75 | 0.69 | 0.66 | 0.62 | 1.00 |  |  |  |  |
| 6 | 0.74 | 0.72 | 0.63 | 0.71 | 0.70 | 1.00 |  |  |  |
| 7 | 0.66 | 0.71 | 0.72 | 0.75 | 0.62 | 0.67 | 1.00 |  |  |
| 8 | 0.79 | 0.72 | 0.73 | 0.78 | 0.72 | 0.73 | 0.84 | 1.00 |  |
| 9 | 0.75 | 0.69 | 0.71 | 0.72 | 0.62 | 0.67 | 0.78 | 0.81 | 1.00 |
